# Retraction Note: Rapid and sustained antidepressant effects of intravenous ketamine in treatment-resistant major depressive disorder and suicidal ideation: a randomized clinical trial

**DOI:** 10.1186/s12888-026-07808-5

**Published:** 2026-01-19

**Authors:** Ahmad Zolghadriha, Afagh Anjomshoaa, Mohammad Reza Jamshidi, Farnaz Taherkhani

**Affiliations:** 1 Department of Psychiatry, Shahid Beheshti Hospital, Zanjan University of Medical Sciences, Zanjan, Iran; 2 Zanjan Metabolic Diseases Research Center, Zanjan University of Medical Sciences, Zanjan, Iran; 3 Department of Anesthesia, School of Medicine, Mousavi Hospital, Zanjan University of Medical Sciences, Zanjan, Iran

**Retraction Note: BMC Psychiatry (2024) 24:341**

10.1186/s12888-024-05716-0

The Editors have retracted this article. Following publication, concerns were raised about the authenticity of the statistical data reported in this paper. The Authors provided some source data on request from the Editors, but an analysis by the Journal found significant discrepancies between this data and the data reported in the article. The Editors have therefore lost confidence in the results and conclusions of this study.

The authors did not explicitly state whether or not they agree with this retraction. 

